# A Natural Language Processing System That Links Medical Terms in Electronic Health Record Notes to Lay Definitions: System Development Using Physician Reviews

**DOI:** 10.2196/jmir.8669

**Published:** 2018-01-22

**Authors:** Jinying Chen, Emily Druhl, Balaji Polepalli Ramesh, Thomas K Houston, Cynthia A Brandt, Donna M Zulman, Varsha G Vimalananda, Samir Malkani, Hong Yu

**Affiliations:** ^1^ Department of Quantitative Health Sciences University of Massachusetts Medical School Worcester, MA United States; ^2^ Bedford Veterans Affairs Medical Center Center for Healthcare Organization and Implementation Research Bedford, MA United States; ^3^ Optum Boston, MA United States; ^4^ Veterans Affairs Connecticut Health Care System West Haven, CT United States; ^5^ Center for Medical Informatics Yale University New Haven, CT United States; ^6^ Division of Primary Care and Population Health Stanford University School of Medicine Stanford, CA United States; ^7^ Veterans Affairs Palo Alto Health Care System Menlo Park, CA United States; ^8^ School of Medicine Boston University Boston, MA United States; ^9^ Diabetes Center of Excellence University of Massachusetts Medical School Worcester, MA United States

**Keywords:** electronic health records, natural language processing, consumer health informatics, usability testing, computer software

## Abstract

**Background:**

Many health care systems now allow patients to access their electronic health record (EHR) notes online through patient portals. Medical jargon in EHR notes can confuse patients, which may interfere with potential benefits of patient access to EHR notes.

**Objective:**

The aim of this study was to develop and evaluate the usability and content quality of NoteAid, a Web-based natural language processing system that links medical terms in EHR notes to lay definitions, that is, definitions easily understood by lay people.

**Methods:**

NoteAid incorporates two core components: CoDeMed, a lexical resource of lay definitions for medical terms, and MedLink, a computational unit that links medical terms to lay definitions. We developed innovative computational methods, including an adapted distant supervision algorithm to prioritize medical terms important for EHR comprehension to facilitate the effort of building CoDeMed. Ten physician domain experts evaluated the user interface and content quality of NoteAid. The evaluation protocol included a cognitive walkthrough session and a postsession questionnaire. Physician feedback sessions were audio-recorded. We used standard content analysis methods to analyze qualitative data from these sessions.

**Results:**

Physician feedback was mixed. Positive feedback on NoteAid included (1) Easy to use, (2) Good visual display, (3) Satisfactory system speed, and (4) Adequate lay definitions. Opportunities for improvement arising from evaluation sessions and feedback included (1) improving the display of definitions for partially matched terms, (2) including more medical terms in CoDeMed, (3) improving the handling of terms whose definitions vary depending on different contexts, and (4) standardizing the scope of definitions for medicines. On the basis of these results, we have improved NoteAid’s user interface and a number of definitions, and added 4502 more definitions in CoDeMed.

**Conclusions:**

Physician evaluation yielded useful feedback for content validation and refinement of this innovative tool that has the potential to improve patient EHR comprehension and experience using patient portals. Future ongoing work will develop algorithms to handle ambiguous medical terms and test and evaluate NoteAid with patients.

## Introduction

### Background and Significance

Enhancing patient access to their clinical data is a central component of patient-centered care [1,2]. In a nationwide effort to reach this goal [3,4], online patient portals have been widely adopted in the United States to allow patients to interact with their personal health care information, including medication lists and laboratory test results from electronic health records (EHRs) [5]. Initiatives such as *OpenNotes* [6] and the Veterans Health Administration’s (VHA’s) Blue Button [7] also allow patients to access their full EHR notes through patient portals, with early evidence showing improved medical comprehension, health care management, and outcomes [8-11].

However, EHR notes are written for documentation and communication between health care providers [12] and contain abundant medical jargon that can confuse patients [13-18]. In addition, an estimated 36% of adult Americans have limited health literacy [19]. Limited health literacy has been identified as one major barrier to patients’ effective use of their EHRs [5,20-22]. Misinterpretation of EHR content may result in patient confusion about their medical conditions and treatment [23], which could potentially impact service utilization, patient satisfaction, or patients’ own self-management [24].

There has been long-standing research in promoting health literacy [25], including the development of online health education resources, for example. However, these methods do not target clinical notes in an EHR. In addition, health information available on the Internet, although abundant, comes from different resources with varied quality and credibility, which poses great challenges to patients in information seeking and selection [26,27]. The readability levels of health information on the Internet are also often greater than that easily understood by average patients [26,28,29].

A few studies of natural language processing (NLP) systems that translate medical terms to lay terms [30,31] or link them to definitions in controlled vocabularies [32] do show improved patient comprehension [30-32]. These NLP-enabled systems have the merits that they provide patients direct help for EHR comprehension by bundling related health information with individual EHR notes. Despite promising results, these methods have some limitations. First, many medical jargon terms do not have associated lay terms (eg, *neurocytoma* and *lymphangiomatosis*). Second, the definitions of medical terms in controlled vocabularies often contain complex concepts that are not self-explanatory. For example, the medical term *GI* is defined in the controlled vocabulary of National Cancer Institute as “A subject domain utilized for the submission of information encompassing and representing data, vocabulary or records related to gastrointestinal system findings,” where the concept *gastrointestinal* may not be familiar to average patients.

### Objective

To address these limitations, we are developing NoteAid, an NLP system that links medical terms in EHR notes to lay definitions targeted at or below the average adult literacy level to support patient EHR comprehension. NoteAid has the potential to be used by veterans supported by VHA, especially the over 2.6 million registered users of Veterans Affairs’s (VA’s) patient portal myHealtheVet [7]. For example, it could be integrated into myHealtheVet as an online tool to help users to understand their clinical notes. Because the challenge in understanding medical terms is not unique to VA patients, NoteAid is also potentially useful for other patient populations. Using a flexible Web-based framework, it can be easily incorporated into patient portals of different health care systems.

In this study, we introduce the main framework of NoteAid and the innovative computational methods we developed to extend its lexical resource and functionality. In addition, as part of the system development procedure, we developed a new evaluation protocol that combines usability testing and quality assessment of lay definitions incorporated into NoteAid by domain experts.

## Methods

### Study Overview

This study presents the NoteAid system and its initial evaluation by physicians. Physicians played the dual role of user and content expert in this usability assessment. Below, we first describe the components and function of NoteAid and then summarize our evaluation protocol.

### The NoteAid System

#### System Overview

**Figure 1:**
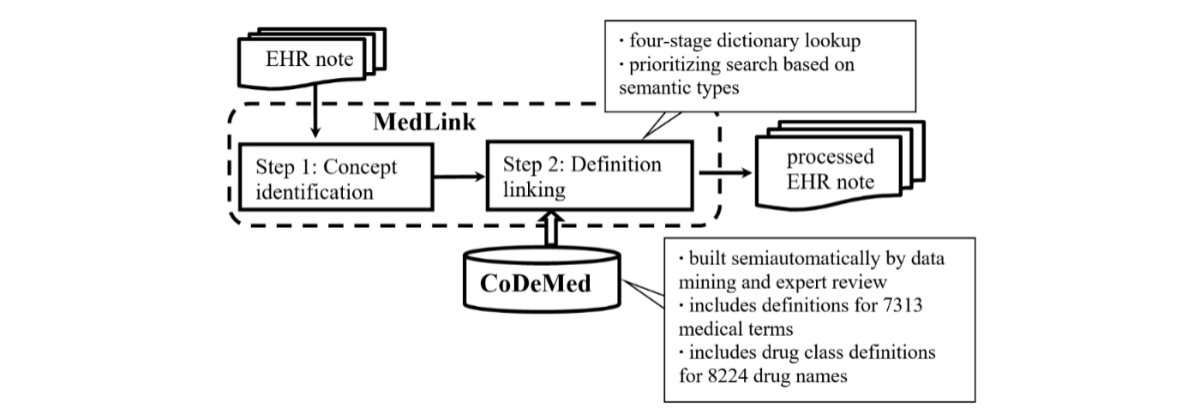
Overview of NoteAid. EHR: electronic health record.

NoteAid is a Web application we developed using Java servlets and JavaScript. Figure 1 shows the NLP components and workflow of NoteAid. NoteAid first identifies medical concepts (step 1) and then links them to lay definitions (step 2). NoteAid builds on two core units: CoDeMed, a lexical resource of lay definitions of medical terms and MedLink, a computational unit that links medical terms to lay definitions. We describe CoDeMed and MedLink in the following two sections by focusing on the computational aspects.

#### CoDeMed

We are developing CoDeMed using both human efforts and automatic methods. By November 2016 when we started this study, CoDeMed contained lay definitions for 7313 medical terms. For example, *Activase* and *bacteremia* were defined as “A drug used to break up blood clots. It is given to patients that have had a stroke or heart attack” and “The presence of bacteria, a type of germ, in the blood,” respectively. In addition, CoDeMed included lay language drug class definitions for 8224 medications that did not have term-level lay definitions yet. For example, *Hecoria* and *Neoral* were mapped to the drug class *calcineurin inhibitors*, which was defined as “A drug used to reduce immune response.”

For quality assurance, all definitions in CoDeMed were collected from authorized online health education resources (eg, glossaries of National Institute of Health and National Cancer Institute) and simplified and reviewed by domain experts, which included MDs. Because this process is time-consuming, we developed an adapted distant supervision (ADS) system to automatically identify important medical terms from EHR corpora to prioritize the annotation efforts on these terms [33].

We defined important terms as those terms that, if understood by patients, would significantly improve their EHR comprehension. In practice, we used four criteria to judge term importance (details in Multimedia Appendix 1).

Instead of using standard supervised learning, we used distant supervision to save manual annotation efforts. Specifically, we used distant supervision from consumer health vocabulary (CHV) [34] by assuming that medical terms important for patient EHR comprehension must represent medical concepts used by patients. Here, we used the broad sense of “patient” to refer to all the health consumers. CHV contains consumer health terms (which were used by lay people to query online health information) and maps these terms to Unified Medical Language System (UMLS) concepts. As a result, it contains both lay terms and medical terms and links between these two types of terms. Our ADS system used medical terms existing in both CHV and an EHR corpus from the University of Pittsburgh NLP Repository [35] (called EHR-Pittsburgh corpus for convenience) as positive examples and used other candidate terms extracted from this EHR corpus as negative examples to train a classification model. For example, the terms *amyloid*, *hypercholesterolemia*, *laminotomy*, and *pulmonary collapse* were among the positive examples, and the candidate terms *admission blood pressure*, *continued pain*, *fainting*, and *lumbar* were among the negative examples. Training data created in this way had noise. For example, important medical terms that do not exist in CHV (eg, *lumbar*) were labeled wrongly as negative examples. To alleviate this problem, our system used transfer learning and a small amount of manually annotated training examples to adapt the classification model to the target domain to identify medical terms that are important for patient EHR comprehension. We empirically show the effectiveness of ADS by using a gold standard dataset of 6038 EHR terms annotated by domain experts [33]. For each candidate term, the ADS system output its probability of being an important term. We used these probability values to rank candidate terms. The top-ranked terms such as *lipodystrophy*, *myelodysplasia*, and *Parkinsonism* from the EHR-Pittsburgh corpus have been incorporated into CoDeMed.

To improve CoDeMed’s coverage, we developed an unsupervised method to mine medical synonyms from Wikipedia [36]. Specifically, we used the interwiki links in pages on the Wikipedia Health tree to extract candidate synonyms for medical terms. For example, *avian influenza* and *bird flu* are linked to the term *avian flu* through the interwiki links, which are both good synonyms of this term; 15 terms are linked to *blood pressure*, which include the synonym (*BP*), the hyponyms (eg, *systemic blood pressure* and *diastolic blood pressure*), and other related terms (eg, *blood pressure measurement* and *low blood pressure*). We then ranked these candidates by using word embedding and pseudo-relevance feedback. Word embeddings are distributed representations of words (which typically are high-dimensional real-valued vectors) learned from large unlabeled text data. Words sharing similar semantics and context are expected to be close in their word vector space [37]. Pseudo-relevance feedback [38] is a method widely used in information retrieval to obtain a better representation of a target concept by using the retrieved results as pseudo-relevance feedback (as opposed to relevance feedback from human annotators). In our case, the target concept is a medical term for which we sought synonyms. We first used other strategies (eg, cosine similarities between the target term and its candidate synonyms) to rank the candidates. We then used the top-ranked candidates to represent the target term and reranked the candidates. We evaluated our methods on 1507 synonyms and nonsynonyms manually judged for 256 medical terms [36]. This method has been used to enrich the candidate synonym set for CoDeMed.

#### MedLink

We developed MedLink to retrieve lay definitions from CoDeMed for medical terms in EHR notes. MedLink utilizes MetaMap [39] to identify medical terms and implements a linking function to retrieve their definitions from CoDeMed.

MetaMap is a widely used lexical tool developed by National Library of Medicine that automatically maps medical text to medical concepts in the UMLS Metathesaurus [39]. For example, both *Cushing’s syndrome* and *Cushing disease* are mapped by MetaMap to the UMLS concept *Cushing Syndrome*.

We have developed two strategies to improve MedLink’s definition linking function. The first strategy is a four-stage dictionary lookup procedure that uses synonym finding and partial string matching to improve coverage of medical terms. For each term identified by MetaMap, MedLink first searches CoDeMed for this term by exact match. If the search fails, MedLink searches for the term’s UMLS preferred name, which is identified by MetaMap. A search failure leads to the third stage, where MedLink shortens the term by removing words in a list (Multimedia Appendix 2) of common modifiers of diseases and body locations, such as *chronic*, *severe*, and *left* and searches the trimmed term. If the search fails again, MedLink searches individual words in the trimmed term and displays lay definitions for every single word that has a hit in CoDeMed. To improve search efficiency, MedLink uses a hash table to store interim search results for reuse.

The second strategy is to speed up the system by prioritizing or deprioritizing EHR terms by their UMLS semantic types. Specifically, we defined 21 prioritized semantic types and three deprioritized ones (Multimedia Appendix 2) by extending our previous work [32] using feedback from domain experts. Prioritized semantic types represent semantic categories of medical concepts commonly used in the clinical domain, such as *Disease or syndrome*, *Pharmacologic substance*, and *Laboratory or test result*. Deprioritized semantic types represent concepts that are too general to have a standalone clinical meaning, such as *Geographic area* and *Temporal concept*. Terms with deprioritized semantic types are ignored (ie, not translated by the system). Terms with prioritized semantic types are searched in CoDeMed using the aforementioned four-stage dictionary lookup procedure. The remaining terms are searched in CoDeMed at only the first two stages of dictionary lookup.

### Evaluation Protocol

#### Content for Evaluation Protocol: Electronic Health Record Notes

The EHR notes used for this evaluation were chosen from the EHR-Pittsburgh corpus because this corpus was deidentified and available for research [35]. Specifically, we randomly selected 200 progress notes from this corpus that satisfied the following two criteria: (1) containing the Assessment and Plan section and (2) containing at least ten medical terms as identified by MetaMap. An expert in public health, who has worked in the civilian and military health care fields for 20 years in the specialties of dermatology, surgery, and emergency medical services reviewed these notes sequentially and selected the first 10 notes whose Assessment and Plan sections include good narratives. Three criteria were used to identify good narratives: (1) the text was written in the conversational tone, as opposed to bulletin points often used in a review of systems or update for other health care providers; (2) the text contained important information about a patient’s diagnoses and treatment plans; and (3) the text was not trivial and contained at least five medical jargon terms. We chose the Assessment and Plan section because this section often contained content that satisfied the first two criteria. We used these sections of the selected notes for system evaluation. The notes we selected were mostly (9 out of 10) intensive care unit (ICU) notes. We chose those notes because they contained abundant complex medical jargon that could be used to challenge the system to test its robustness.

Textbox 1 shows an excerpt from one clinical note used for system evaluation, with a number of medical terms that may hinder patients’ comprehension italicized. Here we show a subset of terms identified by the UMLS lexical tool MetaMap [39] for illustration purposes.

#### Usability Procedures

Our protocol allows physicians to simultaneously assess the system’s user interface and the content quality of lay definitions. This approach is motivated by two factors. First, our system provides patients knowledge of medical terms. The quality of the provided knowledge is an important aspect of its usability. Physicians rather than patients have the proper training to judge the accuracy of definitions for medical terms. Therefore, we asked physicians to evaluate our system at this stage. Second, physicians do not differ from lay people as users of computer software. Here, we used the general meaning of “users of computer software,” that is, people who use a software product without the technical expertise required to fully understand it. From this perspective, we expect physicians to give feedback on user interface (eg, ease of use and speed) in a similar way as patients do.

The evaluation included a 1-hour cognitive walkthrough session and a 7-item postsession questionnaire. Each physician was interviewed separately in the following procedure. At the beginning, the interviewer gave the physician an overview of the system, the assessment procedure, and the goal of the interview—collecting feedback regarding the system’s user interface and output content. In addition, she informed the physician that the system output might not be always accurate and encouraged the physician to seek clarification on definitions they found inaccurate or confusing. She then showed the physician instructions on system use. The physician used the system to process the EHR excerpts one by one, reviewed the output from the system, and gave feedback in a think-aloud manner. The physician was encouraged to make free comments on any aspect of the system.

**Illustration of medical terms in a sample clinical note.**

*Cardiac*—The patient was *hypotensive* yesterday during the day with *pressures* running in the *systolics* of 80's to 90's by *cuff*. *Cardiology* was called to see the patient and they did a quick bedside *echocardiogram* that revealed no *pericardial effusion*. Her *troponins* never went higher than 0.77 and *cardiology* was not concerned with any primary *cardiac event*. Her heart rate was also in the one teens to one twenties.

During this process, the interviewer also asked the physician a few optional questions about the user interface (details in the subsection Physician Responses to Prompts). Except these prompts, the interviewer only observed the physician using the system and responded to his or her questions, without discussing or debating on suggestions for system improvement.

The postsession questionnaire was developed by a group of experts in public health, medicine, health informatics, and computer sciences. Because this study is the first effort to collect physicians’ feedback on the content quality of the NoteAid system, there are no existing validated surveys to use. We therefore elected to develop a short survey with questions that were specific to our system. To ensure the quality of the survey, we asked one clinical domain expert to evaluate the validity of the survey content and asked two lay people to evaluate whether the content is easy to understand. Our questionnaire includes 5 scale questions and 2 optional open-ended questions. The scale questions (details in the subsection Physician Responses to Postsession Questionnaire) evaluate lay definitions in four aspects: readability (Q1), informativeness (Q2), coverage (Q3), and accuracy (Q4 and Q5). The open-ended questions collect free comments on any aspect of the system (details in Multimedia Appendix 3).

We recorded voice, screen, and mouse clicks of the whole interview process, including filling out the survey, by using Morae Recorder (Version 3.3.4., TechSmith Corporation) for data analysis.

#### Participants

A convenience sample of 10 physicians with diverse clinical expertise (details in Table 1) were recruited from Edith Nourse Rogers Memorial Veterans Hospital, VA Palo Alto Health Care System, VA Connecticut Health Care System, and the University of Massachusetts Medical School.

We used a small group of physicians by following previous studies in usability research [40-44] and evaluating clinical NLP systems [30,45-47] and considering factors relevant to our case. The variability of the physicians’ specialty was unintended. Previous work found that, for simple usability tasks, usability testing using 5 to 10 users was able to find over 80% problems, and the cost-benefit ratio of increasing the number of users was high [40-43]. Our task was simple and required the user to do a small, closed set of operations (eg, copying and pasting EHR content into the input box and hitting the “simplify” button to view the output). In addition, our study is a midstage evaluation to prepare the system for late-stage patient evaluations.

#### Data Analysis

We analyzed the audio-recorded think-aloud data and physician responses to open-ended survey questions by qualitative content analysis. Qualitative content analysis is a research method widely used for analyzing written, verbal, or visual communication messages through the systematic process of coding and identifying themes or patterns [48-50]. It has been successfully used to study clinical NLP systems [45,46] and patient’s comprehension of clinical text [14,23]. Following established techniques [49,50], we carried on the analysis over three phases, that is, preparation, organizing, and reporting.

In the preparation phase, two researchers reviewed the think-aloud data from two interview sessions and identified physicians’ comments related to system’s user interface and lay definitions. This review resulted in an initial code book with three broad top-level categories: feedback about the user interface, feedback about the definitions, and other feedback. The first category was further divided into positive comments, system error, and suggested improvements at the second level. The second category was divided into positive comments, inaccurate definitions, suggested improvements, missing definitions, and lay terms that do not need definitions.

In the organizing phase, one researcher coded the data, which were then reviewed by the second researcher. The coder segmented the data, classified the segments by selecting codes (details in Multimedia Appendix 4) from the existing codebook, and created new codes when necessary by discussing with the reviewer. Codes were assigned based on the manifest content of the recordings. Code definitions and coding examples were used to facilitate the coding process (details in Multimedia Appendix 4). 

**Table 1:** Specialty and gender of physicians who participated in NoteAid assessment.

Specialty	Gender	
	Female, n	Male, n
Clinical Pharmacology		1
Endocrinology	1	1
Family Medicine		1
Internal Medicine	2	2
Preventive Medicine	1	
Pulmonology	1	

Few disagreements (4.5% [33/728]) over the coded data were resolved by discussions between the coder and the reviewer. The final coding scheme and categories (ie, themes) formulated over the codes are summarized in Multimedia Appendix 4. To assess intercoder reliability, two researchers independently coded 229 segments from physicians’ think-aloud data, which covered two to four notes randomly selected for each physician. The intercoder agreement on this dataset is .88 Cohen kappa.

In the last phase, we reported descriptive statistics of themes and qualitatively summarized key findings.

## Results

### System Output

**Figure 2:**
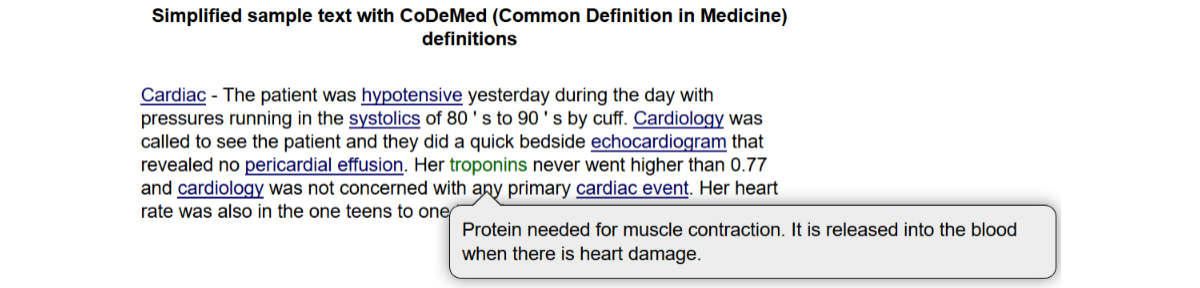
A snapshot of system output.

Figure 2 shows a snapshot of NoteAid’s output on the EHR excerpt in Textbox 1, where 8 medical terms were highlighted and linked to lay definitions by the system. The definition of a medical term, for example, *troponins,* will show up when hovering over this term. The definitions output by NoteAid for all the 8 medical terms are listed in Multimedia Appendix 5.

Table 2 summarizes the EHR data used in the evaluation and NoteAid’s output on this data. As shown in Table 2, NoteAid linked 29.2% of the terms identified by MetaMap to lay definitions. The low ratio is caused by two reasons: (1) some medical terms do not yet have lay definitions in CoDeMed and (2) MetaMap identified both medical jargon terms and lay terms (eg, *patient*, *bleeding*, and *elevated*) commonly used in the biomedical domain. Therefore, this ratio value underestimates NoteAid’s recall. In our result analysis, we used medical terms that at least two physicians judged to be missed by NoteAid and medical terms linked by NoteAid to estimate the recall, which was 0.565 (standard deviation 0.164) per note in this study.

### Analysis of Physician Think-Aloud Data

#### Overview

In total, 8 physicians reviewed 10 EHR notes; 2 physicians reviewed 9 notes in their interview sessions and could not stay longer. We asked all the physicians to fill out the postsession questionnaire even if they did not finish all the notes. We used all available think-aloud data for analysis and coded 728 segments including 71 system-related comments, 593 definition-related comments, and 64 other comments.

#### System Related

The system-related segments include 11 positive comments (1.1), 50 comments suggesting improvements (1.2), and 10 system errors (1.3). Textbox 2 shows some examples. The numbers in the round brackets are the codes assigned for these categories, as detailed in Multimedia Appendix 4. In the rest of this paper, we used A#’s (eg, A1, A2) to represent different physicians.

One major suggestion for improvement (25 comments from 7 physicians [A1, A3, A4, A5, A7, A8, and A9]) was to change the display of definitions for partially matched terms. NoteAid currently highlights the full spans of those terms and displays definitions for their component words or subterms, hoping to give patients as much information as possible. For example, *normal sinus rhythm* is highlighted, but only *rhythm* is defined (which is displayed as “[rhythm]: definition text”). However, many physicians thought this was somewhat confusing. A deep analysis of other comments for the user interface suggests the following improvements: (1) showing system status when it is running (A2, A4, A5, and A6), (2) putting the *“* simplify” button under the search box (A2, A4, A5, and A6), (3) making the label of the search box more informative (A4 and A6), and (4) disabling the dangling hyperlinks (which we planned to use to link to educational materials in the future) for now (A2 and A6).

**Table 2:** Statistics of the electronic health record (EHR) data used for evaluation and NoteAid’s output on these data.

Characteristics of NoteAid’s output	Data
Number of EHR^a^ excerpts, N	10
Number of words per EHR excerpt, mean (SD)	383 (169)
Number of terms identified by MetaMap per EHR excerpt, mean (SD)	141 (66)
Number of terms linked to lay definitions by NoteAid per EHR excerpt, mean (SD)	41 (19)
Percentage of terms identified by MetaMap per EHR note that have been linked to lay definitions by NoteAid, mean (SD)	29.2 (2.8)

^b^EHR: electronic health record.

**Examples of physicians’ comments on NoteAid’s user interface. A#’s refer to different physicians. The numbers in round brackets, for example, (1.1), refer to the codes assigned for data categories.**

Positive comments (1.1):

A1: “It's very efficient in terms of linking the term to the definition. It's fast and accurate as well.”

A4: “The system on this first note, the neuro note, is doing a good job.”

A6: “This could be a great product for pre-med or early med students.”

A7: “You picked up the meds (medications) and the drugs well, most of the concepts.”

A10: “Overall I think it's nice how it's done this.”

Comments suggesting improvements (1.2):

A2: “Ok, so in that case, maybe when someone hits it once, give a, like, time bar or something.”

Interviewer: “To let them know it’s working?”

A2: “Yeah.” (1.2.2)

A4: “Again, you’re underlining more than you actually define.” (1.2.1)

A5: “Maybe the simplify button should go right under there.” (1.2.2)

A6: “Oops, what happens when I click on it, it just hops away. Why does it do that?” (1.2.2)

A7: “What is this?”

Interviewer: “I think that’s something they were trying, I’m not sure if it’s going to help or just make people more confused, to have multiple senses.”

A7: “Yeah, that was confusing, actually.” (1.2.2)

#### Definition Related

Among the definition-related comments, 28 were statements about good definitions (2.1), 54 were about inaccurate definitions (2.2), 92 were about improvements of definitions (2.3), 18 were about lay terms that should not be defined (2.4), and 401 were about missed terms (2.5). Textbox 3 shows some examples. As one term can occur in multiple notes and be commented on by different physicians, we further analyzed these data at term level and identified terms using agreement by at least two physicians.

We found that physicians usually passed good definitions silently, resulting in fewer positive comments (see three examples in Textbox 3).

A total of 12 terms (ie, 6.2% of unique terms linked to lay definitions by NoteAid) were judged to be inaccurate, which included 11 ambiguous terms and acronyms whose definitions provided by NoteAid did not fit the specific context. For example, *AC* is defined by NoteAid as “a short-hand name for a chemotherapy combination used to treat breast cancer,” but it was used as an abbreviation of *assist control* in the EHR note. 

One definition (for the term *Valcyte*) was judged to be inaccurate despite the context. This definition was derived from drug class definitions to improve NoteAid’s coverage of medical terms. Specifically, NoteAid treats *Valcyte* as one type of *Purine Nucleosides*, which is defined as “a drug used to treat cold sores, genital herpes, and chicken pox.”

Physicians suggested improving definitions for 20 (10.3%) terms. In particular, they suggested adding information to the definitions for three terms (ie, *sinus rhythm, diuresis*, and *Lantus*) to improve their clarity and specificity. For example, *sinus rhythm* is defined as “Heart rhythm that begins at the upper chamber of the heart” in CoDeMed, whereas physicians wanted us to clarify that this term referred to the normal heart rhythm. Other suggestions include improving the grammar and readability of certain definitions and unifying the style and granularity of the drug definitions.

A total of 91 terms (ie., 14.8% of unique terms in the ten EHR notes that were identified by MetaMap) were judged to need lay definitions but missed by NoteAid, which include 34 multi-word terms (eg, *tidal volume*, *GJ tube*, and *community acquired pneumonia*) and 57 single-word terms (eg, *FiO2*, *macrocytic*, and *reintubated*). The 34 multi-word terms have meanings beyond the simple sum of their component words. For example, knowing the words *community*, *acquired*, and *pneumonia* is not sufficient for understanding the term *community acquired pneumonia*.

Two terms, *felt* and *level*, were linked to lay definitions but were judged by domain experts to be lay terms that did not need definitions.

#### Other Comments

Comments in this category are mostly observations or suggestions to the evaluation. For example, three physicians (A1, A4, and A5) commented that certain notes were too complicated for patients to understand even if we provided lay definitions for medical jargon, and one physician (A5) suggested the inclusion of simpler outpatient notes for future evaluations. Another noticeable pattern is about note comprehension. For example, two physicians (A3 and A4) pointed out that, in addition to showing the definitions of lab measures, explaining the range of the normal lab values would be also important for patient understanding their lab results.

**Examples of physicians’ comments on definitions that NoteAid provided for medical terms. A#’s refer to different physicians. The numbers in the round brackets, for example, (2.1), refer to the codes assigned for data categories. The context for the term wean in the fourth example is “I will continue to wean his FiO2 as tolerated and will attempt a trach-mask trial today.” NoteAid did not link the term hemodynamically in the fifth example to a definition.**

**Term: antibiotics**

Definition: Medicines that attack bacteria.

A5: “I like the focus on bacteria there.” (2.1)

**Term: BUN**

Definition: [full name: blood urea nitrogen] The amount of nitrogen in blood is measured to check how well the kidneys work. The nitrogen comes from urea which is formed by the breakdown of protein in the liver.

A9: “I like how this one talks about how it's measured to check how well the kidneys work...” (2.1)

**Term: hemoglobin**

Definition: A protein in the blood that carries oxygen. It gives blood its red color.

A4: “It talks to people at the level they can understand, and this extra little bit...I feel like that sort of helps you feel like, I get that. It's just well done.” (2.1)

**Term: wean**

Definition: To be taken off a certain drug very slowly.

A2: “Wean here, the definition is to be taken off a certain drug so I would suggest maybe to change it to be taken off something slowly or a certain regimen.” (2.2.2)

**Term: hemodynamically**

A3: “Hemodynamically needs to be defined.” (2.5.1)

**Term: community acquired pneumonia**

Definitions: [[community]]: A group of people. [[pneumonia]]: An infection of the lungs, usually caused by viruses or bacteria.

A4: “Community acquired pneumonia shouldn't be defined as two separate things, there's no point in defining community for people, and CAP is a concept and should be defined together.” (2.5.2)

**Term: felt**

Definition: Feeling happy, mad, or scared.

A5: “I don't think we need to define felt.” (2.4)

**Term: creatinine**

Definition: A waste product made by muscles and cleared from the blood by the kidneys.

A8: “This again should tell the reader clearly that these are tests that are used to monitor how well the kidneys are working.” (2.3.1)

### Physician Responses to Prompts

Table 3 summarizes physician responses to optional prompts, manually labeled as *satisfied* and *suggesting improvements*. Most respondents thought the system’s speed acceptable. One respondent (A9) commented that higher speed would be better for batch processing of many notes at one time. One respondent (A5) suggested showing a progress bar when the system was working. One respondent (A4) suggested disabling the “simplify” button to avoid multiple hitting (which would slow down the system) when the system was processing a note. All respondents liked how NoteAid displays definitions, but one (A4) commented that using hyperlinks could cause confusion. Most respondents thought that the system was easy to use.

### Physician Responses to Postsession Questionnaire

Table 4 summarizes physician responses to scale questions. The average scores for the four aspects of lay definitions generated by NoteAid range between 3.70 and 4.30.

The analysis of physician responses to the open-ended questions (details in Multimedia Appendix 3) showed that physicians would like to see improvements in robustness (A3 and A6) and layout (A7, A9, and A10) of the user interface and quality of certain aspects of definitions (A2, A4, A6, and A8). In addition, two physicians (A4 and A5) suggested using outpatient notes for future system testing.

Among the positive comments, five physicians (A1, A3, A5, A8, and A10) thought that the user interface was very straightforward and easy to use. Two physicians (A1 and A2) liked the good coverage of medical terms, and one physician (A9) appraised the lay language nature of definitions. In addition, three physicians (A4, A6, and A9) appreciated the usefulness of the system.

**Table 3:** Physician responses to optional prompts on user interface. We report the proportion of physicians who were satisfied or suggested improvements when responding to each prompted question. A#’s denote different physicians.

Questions^a^	Satisfied	Suggesting improvement	Example responses
PQ1. Do you feel the system speed is tolerable?	4/7 [A5, A6, A8, and A10]	3/7 [A3, A4, and A9]	A3: “I think it’s ok. I mean, some of them, this one was a long one and it's taking a little longer. But I suppose people would probably figure that out.”
			A5: “If there was a progress bar or something it would be okay.”
			A6: “Yeah, it seems really fast today.”
			A9: “I think it takes a little longer than ideal to process it. Well just cause the longer it takes, especially when people are trying to do multiple things. But it’s not too bad.”
PQ2. Do you like the way that the system displays the definitions?	9/10 [A1, A2, A3, A5, A6, A7, A8, A9, and A10]	1/10 [A4]	A3: “I like it fine. I think it’s very easy to see which terms are defined and to see the definition. It’s pretty straightforward.”
			A4: “Because there's an underline, I want to click on it. If you had it blue but not underlined...I don't know what to tell you guys to do, it definitely says to me “click on me” and if I'm moving quickly as people are, like, I would figure it would but it's a little confusing.”
			A5: “Yeah, yeah I do. Just hovering over with the mouse. It is convenient.”
			A7: “Yeah, they're short and easy to read.”
PQ3. Is it easy for you to find the definitions that were generated by the system?	4/4 [A1, A2, A3, and A7]	0/4	A1: “Definitely, I just have to point the mouse at any word that I'm wondering what it means.”
			A7: “Oh yeah, you just hover, that's not hard.”
PQ4. Do you think the instructions on the web page are easy to follow?	7/8 [A1, A2, A3, A5, A7, A8, and A10]	1/8 [A4]	A1: “Yes, very easy”
			A4: “I didn't follow them, I just did what you told me. So, you haven't labeled the search box, so I would...or ‘into the box below.’ That may be confusing, I don't know...I would probably give some direction like ‘push simplify button.’”
			A10: “Yeah, just copy and paste.”

^a^PQ# refers to the #th prompted question.

**Table 4:** Evaluation scores for scale questions in postsession questionnaire.

Questions	Domain	Scale^a^	Evaluation score Mean (SD)
Q1. The definitions are in lay language (ie, do not contain medical jargon).	Readability	never 1 2 3 4 5 always	3.90 (0.57)
Q2. The definitions provide useful information for comprehending medical jargon in electronic health record (EHR) notes.	Informativeness	never 1 2 3 4 5 always	3.80 (0.63)
Q3. NoteAid has good coverage of lay definitions for medical jargon in EHR notes.	Coverage	disagree 1 2 3 4 5 agree	4.10 (0.74)
Q4. NoteAid links medical jargon to definitions that are correct or appropriate for patients.	Accuracy	seldom 1 2 3 4 5 often	3.70 (0.67)
Q5. NoteAid links medical jargon to incorrect definitions.	Accuracy	often 1 2 3 4 5 seldom	4.30 (0.95)

^a^We used 5-point Likert-style scale questions. For example, “never 1 2 3 4 5 always” refers to never, seldom, sometimes, often, always.

## Discussion

### Principal Findings

We have developed NoteAid, an NLP system that links medical jargon to lay definitions targeted at or below the average adult literacy level and reported a formative evaluation conducted to improve the system. Ten physicians with diverse backgrounds evaluated NoteAid. Overall, physicians were positive about the user interface and the quality of lay definitions, as indicated by their responses to the survey questions and optional prompts.

Building NLP systems that support patient EHR comprehension is challenging. One major reason is the language use characteristics of EHR notes related to clinicians’ writing behavior [51]. For example, clinicians often use shorthand (eg, abbreviations and acronyms) in clinical narratives, which causes great ambiguity in meanings [52,53]. Simple strategies such as the most frequent sense (MFS) heuristic, is not sufficient to resolve such ambiguity (details in the subsection Lay Definitions). Misspellings are common in clinical texts, which reduce the system’s recall on medical terms. Some clinical texts are ungrammatical, for which lexical-level comprehension support is not sufficient. These characteristics also make it harder for patients to comprehend their EHR notes, given that the notes are already abundant with medical terms. Despite these challenges, NoteAid received positive physician feedback, suggesting that this tool has great potential to be useful for patients.

In addition, physicians provided valuable suggestions for system improvement before we started a randomized trial sponsored by VHA, which fulfilled one major goal of this study.

### User Interface

Physicians thought that system speed was satisfactory (although not optimal) and that the system website was easy to navigate. The definitions, which pop up in grey bubbles when the cursor is hovered above a term, were helpful and easily found. Simple changes such as altering the placement of buttons on the website or revising the label of the input box to add clarity were suggested. We have improved the system based on their feedback in five aspects.

First, we fixed the position of the “simplify” button to be right below the input box, so a user can easily find it even when the output text is long.

Second, we added a function to disable and grey out the “simplify” button when the system starts to process the input text and reenable it after the processing completes. This gives users a sense about when the system is working. It also prevents them from hitting the “simplify” button repeatedly, a behavior that can slow down the system.

Third, we modified the introduction text on the user interface and the label of the input box to improve clarity.

Fourth, we disabled dangling hyperlinks in system output.

Fifth, we investigated the system errors that occurred during the evaluation and found that the errors were caused by a design flaw of the original system. Previously, the system segmented an input text into sentences and reopened a subservice of MetaMap every time it was processing a new sentence. Consequently, the temporary files opened for communication with the subservice sometimes accumulated too quickly to be tolerated by the operating system, causing a system crash. We have fixed this problem by modifying the code to process the input text without sentence segmentation. This change also improved the system speed.

We provide screenshots of the original system and the enhanced one in Multimedia Appendix 6.

Our system has the potential to be integrated into VA’s patient portal after patient testing and further improvement. It can also be used as a standalone tool. Using our system only requires a few simple operations; therefore, we expect it to be easily used by patients with different levels of computer skills. We implemented the system using Hypertext Transfer Protocol Secure that encrypts user page requests and responses from the Web server, to protect patients’ private information.

### Lay Definitions

Physicians often passed good definitions silently. Their positive comments suggest that they liked our approach for defining medical jargon—giving essential information in lay language while omitting other details that could easily overwhelm the patients. This approach helps reduce patients’ cognitive load when they read long EHR notes abundant with medical terms.

Furthermore, physicians gave valuable feedback about which medical concepts are better understood as phrases versus the sum of their individual terms. We have used their feedback to improve our guideline on selecting compound medical terms for inclusion into CoDeMed. This improvement will reduce cases where patients think they understand a term like *community-acquired pneumonia* but in fact not, therefore helping them understand their EHR notes more accurately. The compound terms physicians identified can also be used as examples to develop learning-based methods for automated compound term detection, which will be our future work.

They also identified several inaccurate definitions that need immediate attention. The inaccuracies were mostly caused by ambiguity. We identified two major sources of ambiguity by result analysis: (1) drugs that can treat different diseases and (2) acronyms. NoteAid currently uses the MFS heuristic for acronyms, which were judged by domain experts, and outputs all definitions for multi-purpose drugs. However, the evaluation results suggest that MFS is not accurate enough, and showing multiple definitions for a drug name could cause confusion. Improving accuracy is crucial for patient use of NoteAid because inaccurate definitions add confusion to patients, and it would be unlikely for them, especially those patients with low health literacy, to discover such inaccuracies by themselves. In the future, we will address this problem by developing automatic methods to predict senses of ambiguous terms in EHR notes.

From physician’s comments, we observed some disagreement about appropriate length and scope of definitions. Most physicians agreed that longer, more detailed descriptions of medical concepts were preferable; however, one physician (A6) argued for the *less is more* approach, citing the already too complex nature of EHR notes. In addition, some physicians thought that only providing lay definitions to medical terms may not be sufficient for full EHR note comprehension. These comments suggest that patient EHR comprehension is challenging and unlikely to have a one-shot solution. For example, patients at higher literacy levels may prefer more detailed definitions, whereas patients at low levels may find such definitions overwhelming. This feedback suggests that the current NoteAid design may be sufficient to support EHR comprehension, but in some cases additional tools may be required. For a more comprehensive solution, one may extend NoteAid by incorporating definitions at different granularity levels and methods interactively measuring patients’ literacy levels.

Drug definitions were another source of disagreement between physician reviewers. Although some preferred simple, all-encompassing definitions (eg, all antibiotics having the same brief definition, “ *Flagyl: a drug that kills germs* ”), others felt more detail should be provided about specific conditions each drug is used to treat. One way to resolve the discrepancy is to introduce hierarchical definitions that show the simple generic definition first and then details specific to context.

Physicians identified many terms which do not present in CoDeMed and thus were missed by NoteAid. We have added 4502 more definitions in CoDeMed since this evaluation, which improved NoteAid’s recall of medical terms in the ten EHR excerpts used for this study from 0.565 to 0.787. We have also developed a hybrid strategy that uses the UMLS controlled vocabularies to back up CoDeMed for missed terms while we simultaneously continue to improve its coverage. We expect that including more lay definitions will benefit patients, especially those with low health literacy.

### Related Works

In the Introduction section, we have discussed the difference of our system from previous systems [30-32] in supporting patient EHR comprehension. Our system is the first one that links medical terms to lay definitions in EHR notes. Our system used drug class definitions for medications that did not have term-level definitions in CoDeMed, which shared the same spirit with the method of using UMLS relations (eg, “Is-A” relation) to generate general explanations for medical terms that lack corresponding lay terms [30].

Previous work in evaluating NLP systems supporting patient EHR comprehension focused on their effects on comprehension [30-32] and sometimes cleaned system outputs manually before presenting them to users [30]. The purpose of our evaluation is different. We evaluated both the user interface and content quality of the system’s original output as part of our development effort to prepare the system for a late-stage patient evaluation.

Similar to previous work in evaluating clinical NLP systems [30,45-47], we used task-based evaluation. The think-aloud walkthrough evaluation protocol used in our study is similar to previous work in evaluating online search engines in answering physicians’ questions [45] and clinical NLP software [46]. Zheng et al [46] found that clinical NLP software that required installation before use often presented challenges to end users. Compatible with their findings, our system, which adopts a Web-based framework without installation requirement on the user side, received positive feedback in terms of ease of use.

### Limitations

Similar to previous work in usability testing of clinical NLP systems, we obtained feedback about NoteAid from a small group of users. Usability testing identified important issues for system improvement, although a larger scale evaluation may yield additional refinements. We used notes from ICU patients, which may not be representative of notes for patients at lower acuity, including individuals seen in outpatient clinics. However, because these notes often cover complex clinical issues, they offer appropriate material to test the system’s robustness. We plan to continue validation of NoteAid using other types of notes, including outpatient notes. We evaluated our system by using physician reviews to get feedbacks on the quality of lay definitions. Physicians, who have received high education, might have better computer skills than people who rarely used computers. We will include patients with mixed levels of computer skills in our patient study in the future.

### Conclusions

Physician evaluation yielded positive results and useful feedback for content validation and refinement of this innovative tool. We have improved NoteAid based on physicians’ feedback. Next steps include a study engaging patients to test the system. Tools such as NoteAid may have the potential to improve patient EHR comprehension, which, when used concurrently with patient portals such as VA’s MyHealtheVet [7,54], can improve patient experience, engagement, and health knowledge.

## Appendix

Multimedia Appendix 1 Criteria for judging importance of candidate terms extracted from electronic health record (EHR) corpora.

Multimedia Appendix 2 Auxiliary data used by NoteAid for medical concept extraction.

Multimedia Appendix 3 Physician responses to open-ended questions in postsession questionnaire.

Multimedia Appendix 4 Coding scheme for qualitative content analysis.

Multimedia Appendix 5 Lay definitions output by NoteAid for medical terms in an electronic health record note excerpt.

Multimedia Appendix 6 User interfaces of the original NoteAid system used in this study and the system with postevaluation enhancements.

## Abbreviations

ADS: adapted distant supervision
CHV: consumer health vocabulary
EHR: electronic health record
ICU: intensive care unit
MFS: most frequent sense
NLP: natural language processing
UMLS: Unified Medical Language System
VA: Veterans Affairs
VHA: Veterans Health Administration
