# Supplementary material for: A Natural Language Processing System That Links Medical Terms in Electronic Health Record Notes to Lay Definitions: System Development Using Physician Reviews
Source: J Med Internet Res. 2018 Jan 22;20(1):e26. doi: 10.2196/jmir.8669 (PMC5799720; doi:10.2196/jmir.8669)
Supplement: Multimedia Appendix 2 [file jmir_v20i1e26_app2.pdf]

Multimedia Appendix 2. Auxiliary data used by NoteAid for medical concept extraction.

Table A2-1. Words removed from medical terms.

Words removed from terms	Example terms (original form → transformed form)
left, right, bilateral, acute, chronic, severe, low, mild, high, history of, flash	left femur → femur<br>acute gastritis → gastritis<br>high systolic blood pressure → systolic blood pressure

Table A2-2. Semantic types used for prioritizing or deprioritizing medical concepts.

Prioritized UMLS semantic types (example terms)	De-prioritized UMLS semantic types (example terms)
Acquired abnormality<br>(anal stricture, colostomy)	Conceptual entity<br>(treatment, home, history, Dr.)
Antibiotic<br>(gentamicin, Keflex)	Geographic area<br>(community, Hawaii, highlands)
Body part, organ, or organ component<br>(venous, sigmoid colon)	Temporal concept<br>(delayed, hourly, long-term)
Cell or molecular dysfunction<br>(mutation, concentric hypertrophy)	
Clinical attribute<br>(visual acuity, vital signs)	
Clinical drug<br>(Flonase nasal spray, nitroglycerin pill)	
Diagnostic procedure<br>(Doppler, transthoracic echocardiogram)	
Disease or syndrome<br>(hydronephrosis, idiopathic myelofibrosis)	
Experimental model of disease<br>(cancer model, knock-out)	
Finding<br>(diabetic, hypesthesia)	

Health care activity<br>(critical care, health screening)
Laboratory procedure<br>(culture, complete blood count)
Laboratory or test result<br>(motility, urine creatinine)
Neoplastic process<br>(metastasis, myelofibrosis)
Organ or tissue function<br>(heart rhythm, diuresis)
Organic chemical<br>(bupropion, procainamide)
Pathologic function<br>(fibrosis, hemorrhage)
Pharmacologic Substance<br>(oxaliplatin, idoxifene)
Physiologic function<br>(coagulation, ventilation)
Sign or symptom<br>(hypokinetic, tenderness)
Therapeutic or preventive procedure<br>(ablation, chemotherapy)
