# Supplementary material for: A Natural Language Processing System That Links Medical Terms in Electronic Health Record Notes to Lay Definitions: System Development Using Physician Reviews
Source: J Med Internet Res. 2018 Jan 22;20(1):e26. doi: 10.2196/jmir.8669 (PMC5799720; doi:10.2196/jmir.8669)
Supplement: Multimedia Appendix 3 [file jmir_v20i1e26_app3.pdf]

Multimedia Appendix 3. Physician responses to open-ended questions in post-session questionnaire.

Table A3-1. Physician responses to open-ended questions in post-session questionnaire. The numbers in columns 3-4 are the codes assigned to data categories during qualitative content analysis, as detailed in Table A4-1 in Multimedia Appendix 4. A#'s (e.g., A1, A2) represent different physicians.

Questions	Answers	User Interface	Definition	Other
Q6. (optional) If there is one thing you could change about NoteAid to make it better, what would it be?	A2: Define terms in a more general way		2.3.2	
	A3: Sometimes it didn't translate the entire note	1.3		
	A4: There's probably a big difference between use of this for routine outpatient notes compared to those tested here - highly complex inpatient ICU notes. Changes: improve use of text at lower literacy levels, pick up more terms.		2.3.2; 2.5	3
	A5: If planning to use this for outpatient care notes, it would be important to have physicians and patients at some point review outpatient care notes. Many words used frequently in ICU notes will almost never appear in outpatient care notes, and vice versa.			3
	A6: It crashed during the test. I would also make the definitions more consistent.	1.3	2.3.2	
	A7: would like to have original text visible next to annotated text, and mechanism to correct/give feedback in note	1.2		
	A8: Definitions should be in the context of the note		2.2.2	
	A9: I think the layout could be improved to make it more accessible to individuals with vision loss, etc. Increase font size and spacing so that the hyperlinks are not so close to one another	1.2		
	A10: Improve consistency of the terms highlighted. For example, PICC line, central line are highlighted but not A line. Or another example is the species of bacteria is sometimes highlight but not in other instances.	1.2	2.5	
Q7. (optional)	A1: I like the speed in how the definitions	1.1	2.1	

What is the one thing you like most about NoteAid?	show up when the term is selected. It covers most of the terms that need definitions			
	A2: It has a very decent coverage.		2.1	
	A3: It is very easy to use. Instructions and interface are very clear.	1.1		
	A4: Ambitious and important effort. Keep up your hard work :)			3
	A5: Easy to use, straightforward interface	1.1		
	A6: I learned a new word!			3
	A8: Simple to use	1.1		
	A9: Provides patients with basic definitions for common jargon, thereby making their medical notes more accessible.		2.1	3
	A10: Simplicity in use and interface.	1.1		
