# Supplementary material for: A Natural Language Processing System That Links Medical Terms in Electronic Health Record Notes to Lay Definitions: System Development Using Physician Reviews
Source: J Med Internet Res. 2018 Jan 22;20(1):e26. doi: 10.2196/jmir.8669 (PMC5799720; doi:10.2196/jmir.8669)
Supplement: Multimedia Appendix 4 [file jmir_v20i1e26_app4.pdf]

Multimedia Appendix 4. Coding scheme for qualitative content analysis.

Table A4-1. Coding scheme for qualitative content analysis.

Label/Name	Definition
1. System related	Comments that form significant themes related to system's user interface
1.1 Positive comments	Appraising speed, ease of use, or other aspects of user interface
1.2 Suggested improvements	Suggesting improvements of user interface
1.2.1 Improving display of partial definitions for phrases	Improving the way of displaying definitions when only part of a phrase was defined
1.2.2 Other aspects of user interface	Improving other aspects of user interface, such as the position of the "simplify" button and the clarity of the input box label
1.3 System error	System did not respond or failed to process the complete input text
2. Definition related	Comments that form significant themes related to definitions
2.1 Positive comments	Appraising accuracy, lay-language nature, or other aspects of definitions
2.2 Inaccurate definition	The definition was incorrect
2.2.1 Inaccurate without context	The definition itself was incorrect
2.2.2 Inaccurate in specific context	The definition did not fit a specific context
2.3 Suggested improvements	The definition was accurate, but the reviewer suggested to improve the definition
2.3.1 Adding specific information	Adding specific information to the definition to improve its quality
2.3.2 Other refinements	Refining other aspects, e.g., grammar and simplicity, of the definition
2.4 Lay terms defined	Terms that are easy to understand were defined
2.5 Terms Missed	Medical terms that need definitions were missed by the system

2.5.1 Completely missed	Missed a whole term
2.5.2 Partially missed	Defined only part of a compound (multi-word) term
3. Other comments	Comments not forming significant themes related to definitions or user interface

Codes were assigned to segments based on the manifest content of the recordings, which included reviewers' comments, their cursor positions and movements, and the system output. The definitions of codes were used as the inclusion and exclusion criteria to assign codes. In addition, we used coding examples to help clarify difficult cases (see some examples below) and used peered review to ensure quality of data analysis.

Example 1: "NPH insulin is the wrong definition for NPH. Someone's probably mentioned that before."
Category: 2.2.2 (Inaccurate definition in specific context)
Reason: The reviewer did not say that the definition was wrong in this specific context. However, the definition that the system displayed was correct without context, which indicated that the definition was wrong in this specific context. Here, both word cues and screen cues (i.e., the term's definition, the context where this term occurred) were used.

Example 2: "it's just weird when you highlight a big long term, but then only define part of it."
Category: 1.2.1 (Improving display of partial definitions for phrases)
Reason: The focus of the comment is about the display.

Example 3: "Antimicrobial therapy is worth putting together as one concept"
Category: 2.5.2 (Partially missed phrase definition)
Reason: The focus of the comment is about the definition. The reviewer suggested to definine the whole phrase rather than its individual words.

Example 4: "You need to clarify that sinus rhythm is a normal thing"
Category: 2.3.1 (Add specific information to definition)
Reason: The reviewer suggested to add specific information ("sinus rhythm is a normal thing") to the definition.
