# Supplementary material for: A Natural Language Processing System That Links Medical Terms in Electronic Health Record Notes to Lay Definitions: System Development Using Physician Reviews
Source: J Med Internet Res. 2018 Jan 22;20(1):e26. doi: 10.2196/jmir.8669 (PMC5799720; doi:10.2196/jmir.8669)
Supplement: Multimedia Appendix 5 [file jmir_v20i1e26_app5.pdf]

Multimedia Appendix 5. Lay definitions output by NoteAid for medical terms in an electronic health record note excerpt

Figure A5-1. A snapshot of system output.

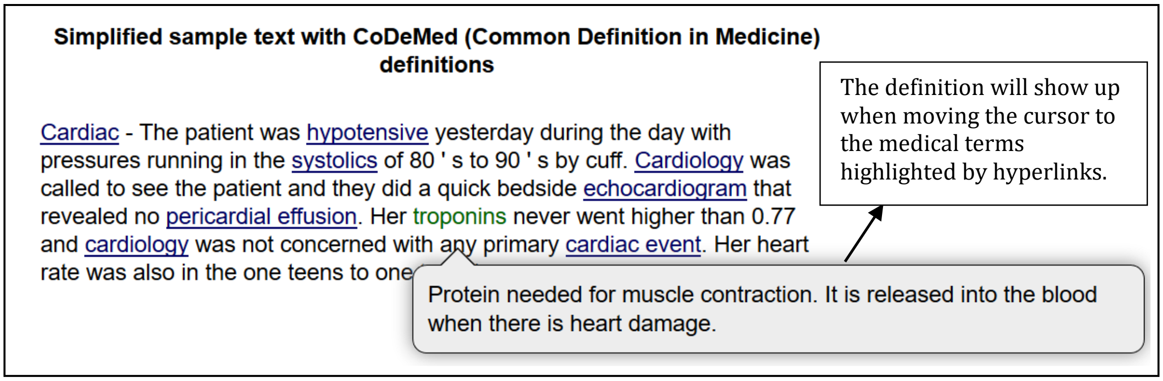

Table A5-1. Lay definitions output by NoteAid for medical terms in the electronic health record excerpt in Figure A5-1.

Medical term	Lay Definition
cardiac	Having to do with the heart.
hypotensive	Having low blood pressure.
systolics	The top number in a blood pressure reading. It refers to the pressure in the arteries during heart muscle contraction.
cardiology	Medical field that treats heart problems.
echocardiogram	Picture of the heart using sound waves (ultrasound).
pericardial effusion	A condition in which extra fluid collects between the heart and the sac around the heart.
troponins	Protein needed for muscle contraction. It is released into the blood when there is heart damage.
cardiac event	[[cardiac]]: having to do with the heart.
