# Supplementary material for: A Natural Language Processing System That Links Medical Terms in Electronic Health Record Notes to Lay Definitions: System Development Using Physician Reviews
Source: J Med Internet Res. 2018 Jan 22;20(1):e26. doi: 10.2196/jmir.8669 (PMC5799720; doi:10.2196/jmir.8669)
Supplement: Multimedia Appendix 6 [file jmir_v20i1e26_app6.pdf]

Multimedia Appendix 6. User interfaces of the original NoteAid system used in this study and the system with post-evaluation enhancements

Figure A6-1. Comparison of user interfaces of the original NoteAid system and the enhanced system when they are processing the input text

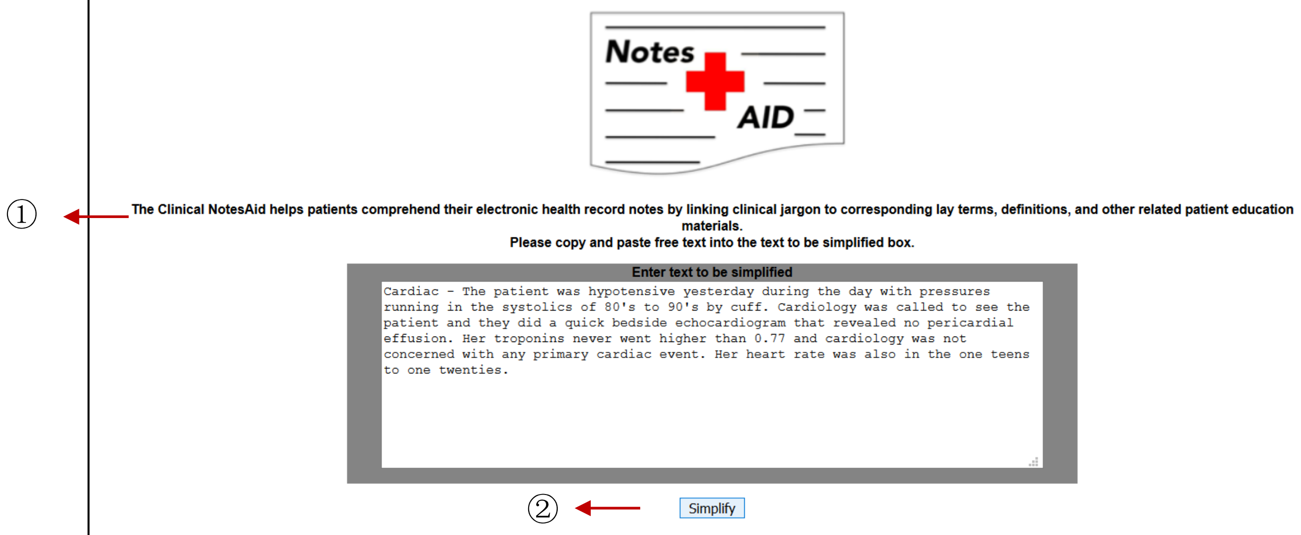

(a) original system

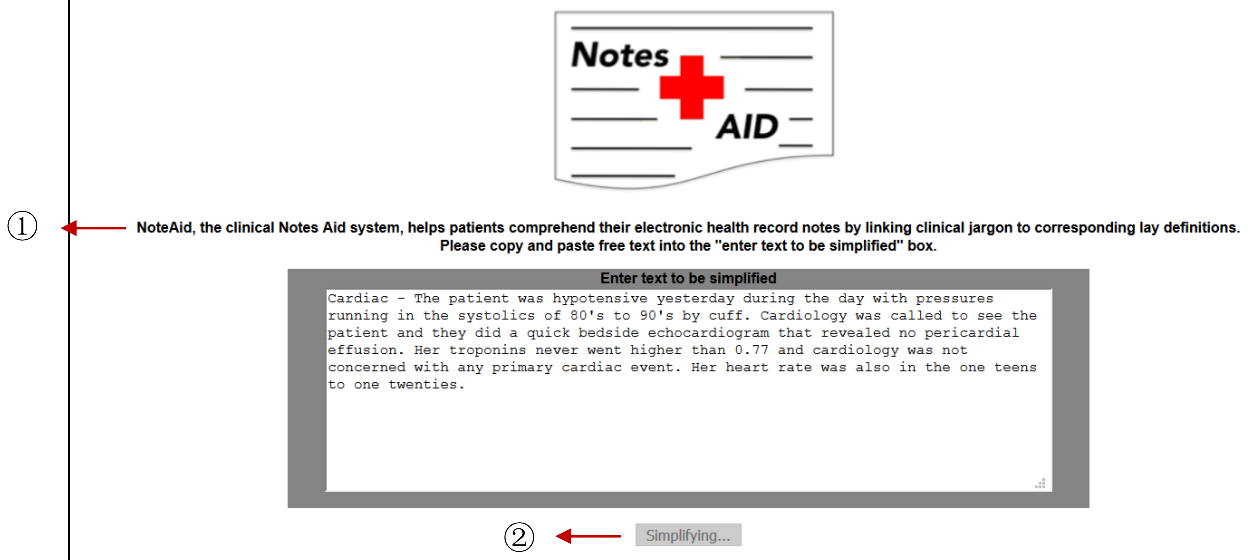

(b) enhanced system

Notes:

① The new system improved clarify of the introduction text for the NoteAid system

② The new system disables and greys out the "simplify" button when the system starts to process the input text and re-enables it after the processing completes

Figure A6-2. Comparison of the original NoteAid system and the enhanced system in displaying system output

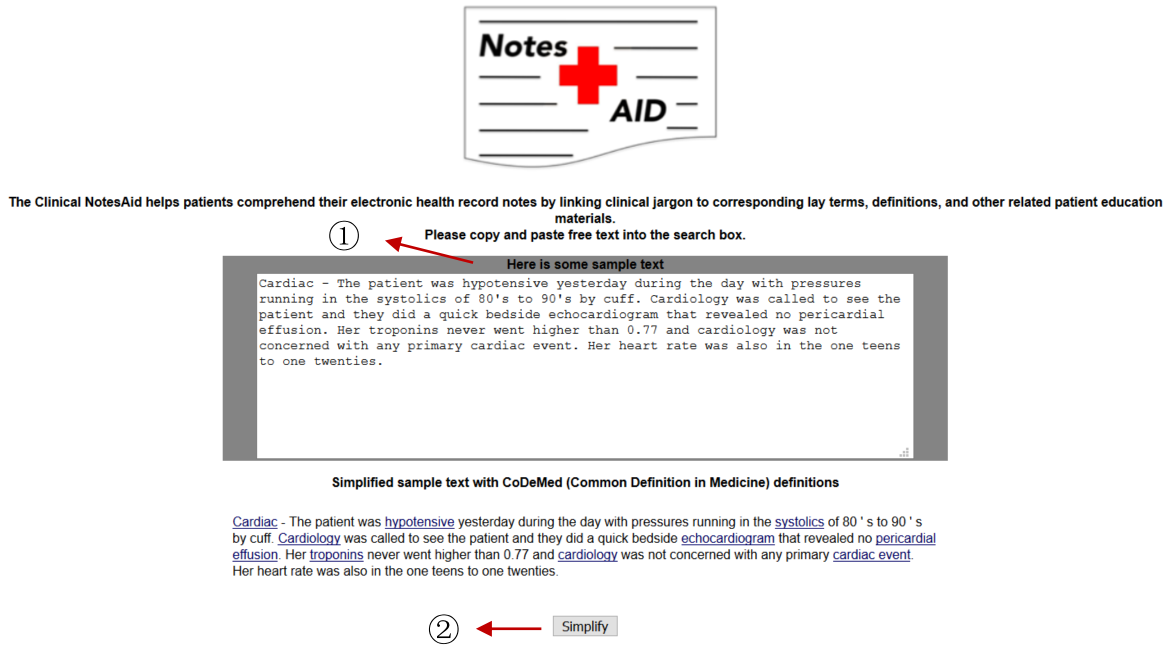

(a) original system

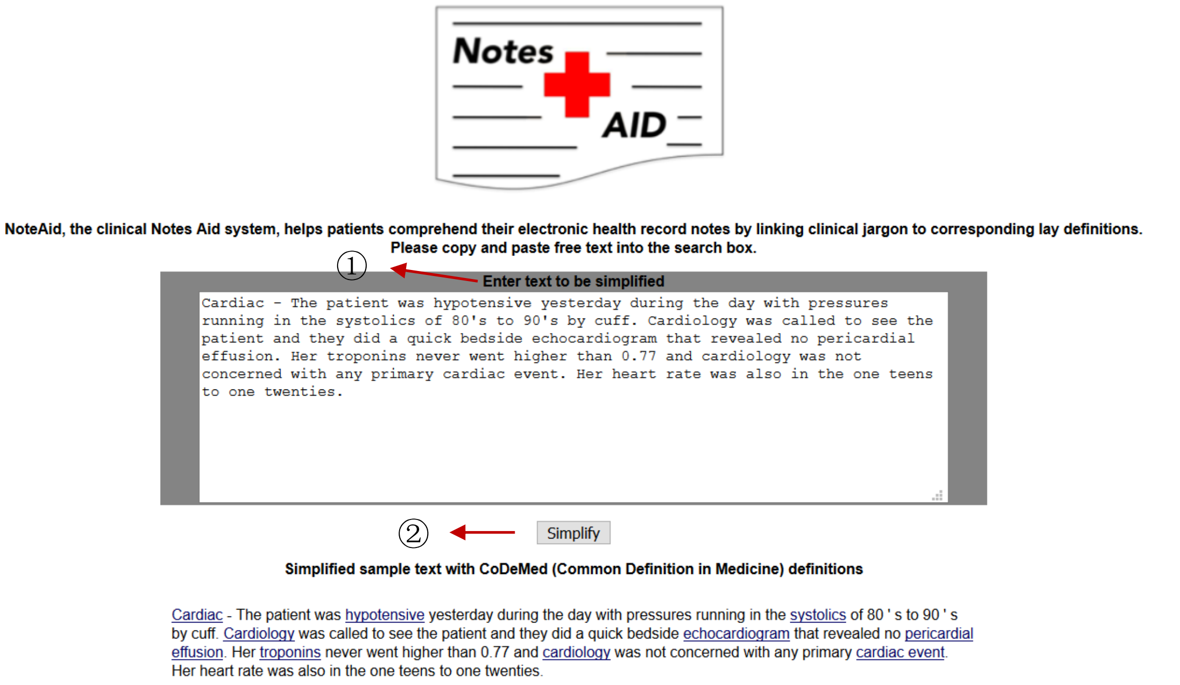

(b) enhanced system

Notes:

① The new system improved clarify of the input box label

② The new system keeps the simplify button right below the input box
